# Imaging Findings of Small Bowel Diverticulitis: A Case Report

**DOI:** 10.21980/J8F078

**Published:** 2023-01-31

**Authors:** Albert Zhou, Sarah Bella, Amy Patwa

**Affiliations:** * Atlantic Health System, Morristown Medical Center, Morristown, NJ

## Abstract

Small bowel diverticulitis is an uncommon subset of acute diverticulitis and can mimic many other intra-abdominal processes. As a result, imaging modalities such as ultrasound and computed tomography (CT) scan are especially important for timely recognition of diverticulitis and can expedite diagnosis and treatment and reduce complications. In the case described in this report, an 81-year-old male with history of esophageal cancer and recurrent diverticulitis with history of multiple bowel resections presented to the emergency department (ED) with right lower quadrant abdominal pain and constipation. Findings on ultrasound were suggestive of diverticulitis, and findings on CT of the abdomen and pelvis showed ileitis with phlegmon and micro-abscess suspicious for small bowel diverticulitis. ED providers should familiarize themselves with ultrasound findings of diverticulitis and be aware that diverticulitis can also present in the small bowel. Treatment of small bowel diverticulitis is similar to colonic diverticulitis.

**Topics:**

Ileitis, small bowel diverticulitis, abdominal ultrasound.[Fig f1-jetem-8-1-v1][Fig f2-jetem-8-1-v1][Fig f3-jetem-8-1-v1]

**Figure f1-jetem-8-1-v1:**
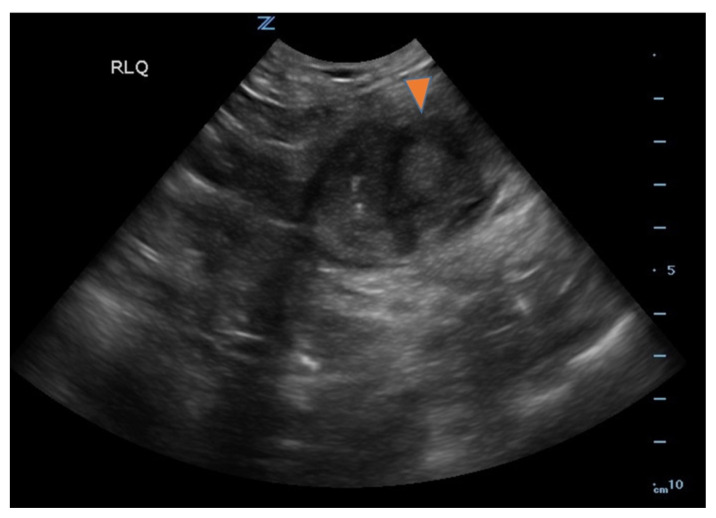


**Figure f2-jetem-8-1-v1:**
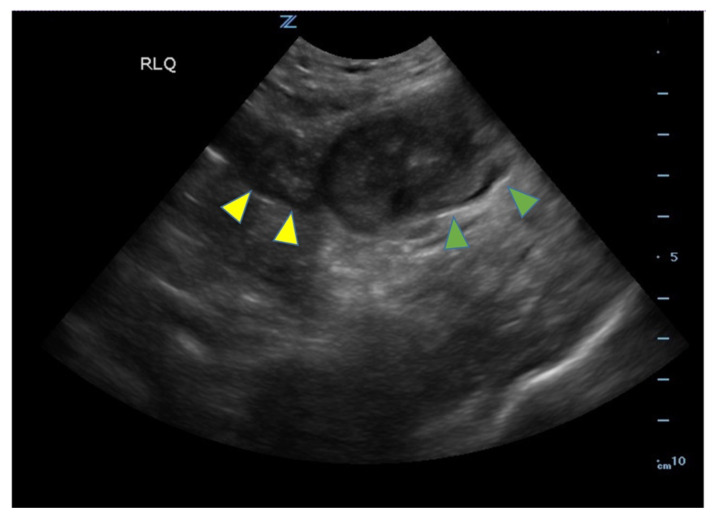


**Figure f3-jetem-8-1-v1:**
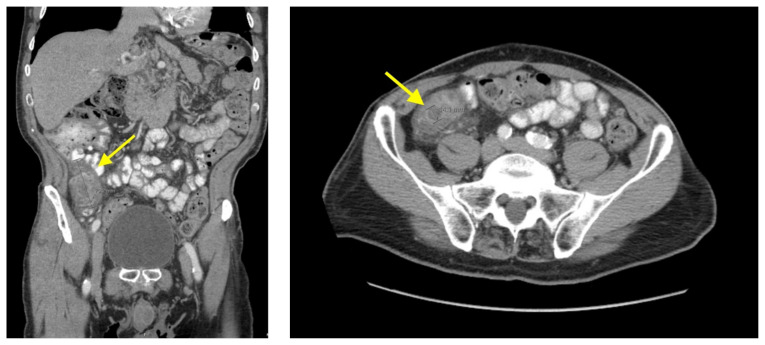


## Brief introduction

Diverticula are small sac-like protrusions in any part of the gastrointestinal tract. Most commonly, diverticula form in the large intestine; however, they can also form in the small intestine, with most being found in the duodenum, and fewer being found in the jejunum or ileum. The majority of patients with small intestinal diverticula are male, and aged >50 years.^1^ When stool is trapped in diverticula, this can lead to inflammation and infection, resulting in diverticulitis. Diverticular disease is the third most common gastrointestinal illness that requires hospitalization and is the leading cause of elective colon resection.^2^ Thus, in older patients presenting with abdominal pain and changes to bowel movements, diverticulitis should always be considered as a potential diagnosis.

This report focuses on the ultrasound and computed tomography (CT) scan findings of small bowel diverticulitis. Ultrasound can be used by providers at bedside, and findings are similar to that of large bowel diverticulitis. They include hypoechoic thickening of the intestinal wall and hyperechoic tissue around diverticula, representing inflammation of fat.^3^,^4^ Typical findings on CT include a mass lesion with extraluminal gas, mural edema of adjacent small bowel loops, and inflammatory mesenteric infiltration.^5^

## Presenting concerns and clinical findings

An 81-year-old male with history of esophageal cancer and recurrent diverticulitis requiring multiple bowel resections presented to the emergency department (ED) for right lower quadrant pain that woke him up from sleep the day prior. He stated that the pain was constant, cramping, and radiated across his lower abdomen. He denied having any nausea, vomiting and reported that he was passing gas and having bowel movements, but had been feeling constipated. His abdomen was non-distended, but he was noted to have a tenderness in the right lower quadrant. Bedside ultrasound was initially performed followed by CT scan of the abdomen and pelvis. Significant labs include WBC of 10.7 k/mm^3, Hgb of 12.6 g/dL, and Lactate of 2.6 mmol/L.

## Significant findings

Bedside ultrasound was performed and showed thickened bowel wall (orange marker), fat enhancement (green marker), and phlegmonous structure with central echogenicity (yellow marker). Imaging of the abdomen and pelvis with CT showed marked wall thickening and inflammatory change involving a 7.0cm segment of the distal/terminal ileum suspicious for severe ileitis with phlegmon and microabscess on the coronal image (yellow arrow). Additonally, the transverse images show a small rim-enhancing focus within this region of inflammation measuring up to 1.4cm which could represent microabscess (yellow arrow). Diagnosis of diverticulitis by ultrasound is made by identifying the following findings: colon wall thicker than 5mm, fat enhancement, evidence of abscess, visualized diverticuli, air artifacts suggesting diverticuli, and tenderness with compression of the probe.^6^ Diagnosis of diverticulitis by CT is made by identifying the following findings: colonic wall thickening, pericolic fat stranding, abscess formation and enhancement of the colonic wall. Often, these signs are associated with an identifiable inflamed diverticulum.^7^

## Patient course

Diagnostic methods of diverticulitis include ultrasound, CT, barium enema, and magnetic resonance imaging (MRI). Although CT scan is widely regarded as the gold standard for diagnosing diverticulitis, a meta-analysis found that evidence for MRI and CT as a diagnostic method of choice was poor, while US showed acceptable accuracy for clinical use.^8^ In this case, both CT scan and ultrasound were performed to determine the diagnosis. After the diagnosis was determined, the patient was treated in the ED with crystalloid fluids and antibiotics. Surgical consult was obtained, and medical admission was advised in addition to gastroenterology consult, nothing by mouth (NPO) status, and continuation of intravenous (IV) fluids and antibiotics. The patient was evaluated by gastroenterology, who recommended low residue diet, bowel regimen, and esophagogastroduodenoscopy **(**EGD) and colonoscopy as an outpatient due to concern for recurrent small bowel diverticulitis. Over the course of his hospital stay, his pain improved and his diet was advanced as tolerated. He was discharged on oral antibiotics.

## Discussion

Diverticular disease and its complications are highly prevalent in the American population, and annually, there are over 200,000 inpatient admissions for diverticulitis.^9^ The most common complications of diverticulitis are phlegmon or abscess, with mortality highest in patient with perforation or abscess.^10^ Although diverticula and diverticulitis are common in the colon, they are rare in the small bowel and particularly rare in the ileum. Presenting symptoms of small bowel diverticulitis can vary widely and can mimic those of appendicitis. Thus, imaging studies are especially important in diagnosis. For large bowel diverticulitis, the diagnostic criteria for both ultrasound and CT are the same: 1) at least one diverticulum, 2) signs of inflammation of fat and 3) thickened bowel wall. A recent prospective study evaluating point-of-care ultrasound in the ED found that ultrasound had a sensitivity of 92% and specificity of 97% in the diagnosis of diverticulitis.^11^ However, other studies found that ultrasound was inferior to and had lower sensitivity and specificity as compared with CT scan,^12^ which has been shown to have sensitivity and specificity of 97% and 98%, respectively, and can also diagnose abscess and contained perforations with high accuracy that may be difficult to appreciate with ultrasound.^13^ Sensitivity and specificity for colonic diverticulitis of bowel wall thickening, fat stranding, and phlegmon with CT scan are 96% and 91%, 95% and 90%, and 4% and 100%, respectively.^14^ There is a paucity of literature describing the sensitivity and specificity of imaging findings of small bowel diverticulitis on CT and ultrasound. Although ultrasound may have lower sensitivity in ruling out diverticulitis compared to CT, it may be appropriate as the initial and only imaging test in cases where there is high suspicion for uncomplicated diverticulitis, eg, in patients with history of diverticulitis that have minimal abdominal tenderness and no signs of systemic infection. These patients could be treated empirically with antibiotics, even if ultrasound is negative.

Emergency medicine providers should be aware that although uncommon, acute diverticulitis can also occur in the small bowel and present with symptoms similar to other intra-abdominal processes. There are no specific guidelines for treatment of small bowel diverticulitis. However, in practice, treatment of colonic diverticulitis can be used as a guide, which in uncomplicated cases includes bowel rest, pain control, and antibiotics.^15^ For complicated diverticulitis, emergency surgery is indicated for sepsis or peritonitis, and urgent surgery is performed if a patient’s condition fails to improve with medical therapy, or if percutaneous drainage is unsuccessful.^16^ Point of care ultrasound is a reasonable initial imaging modality to diagnose acute diverticulitis, but CT should be considered when abscess is suspected. Small bowel diverticulitis should be considered in the differential diagnosis of acute abdominal pain, especially in elderly patients because timely recognition and management of patients with small bowel diverticulitis can reduce morbidity and mortality, and reduce incidence of complicated diverticulitis.

## Supplementary Information
















