# Supplementary figures and images for: Imaging Findings of Small Bowel Diverticulitis: A Case Report
Source: J Educ Teach Emerg Med. 2023 Jan 31;8(1):V1–4. doi: 10.21980/J8F078 (PMC10332775; doi:10.21980/J8F078)

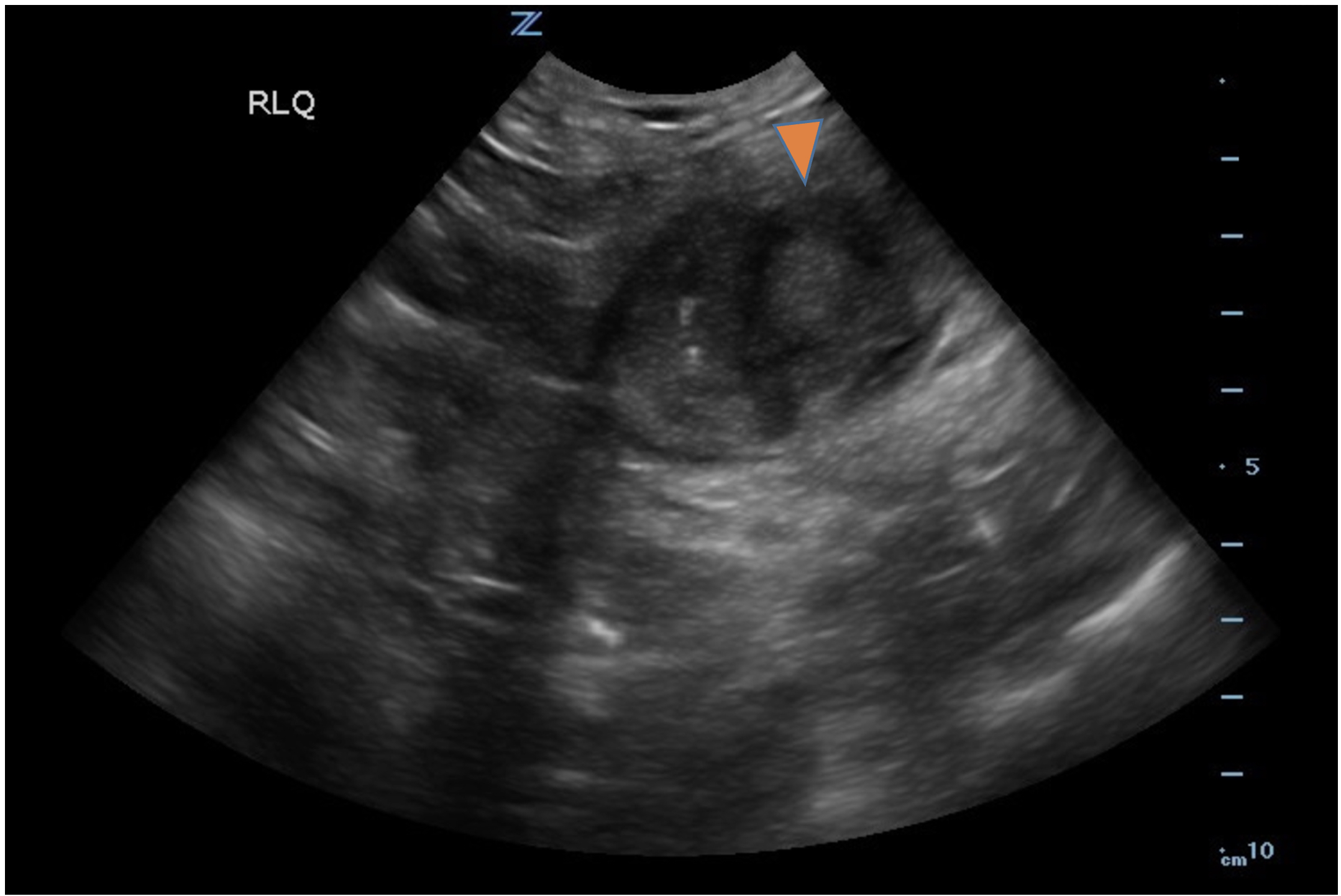

Supplement: Supplementary file 1 [file jetem-8-1-v1-supp1.jpg]

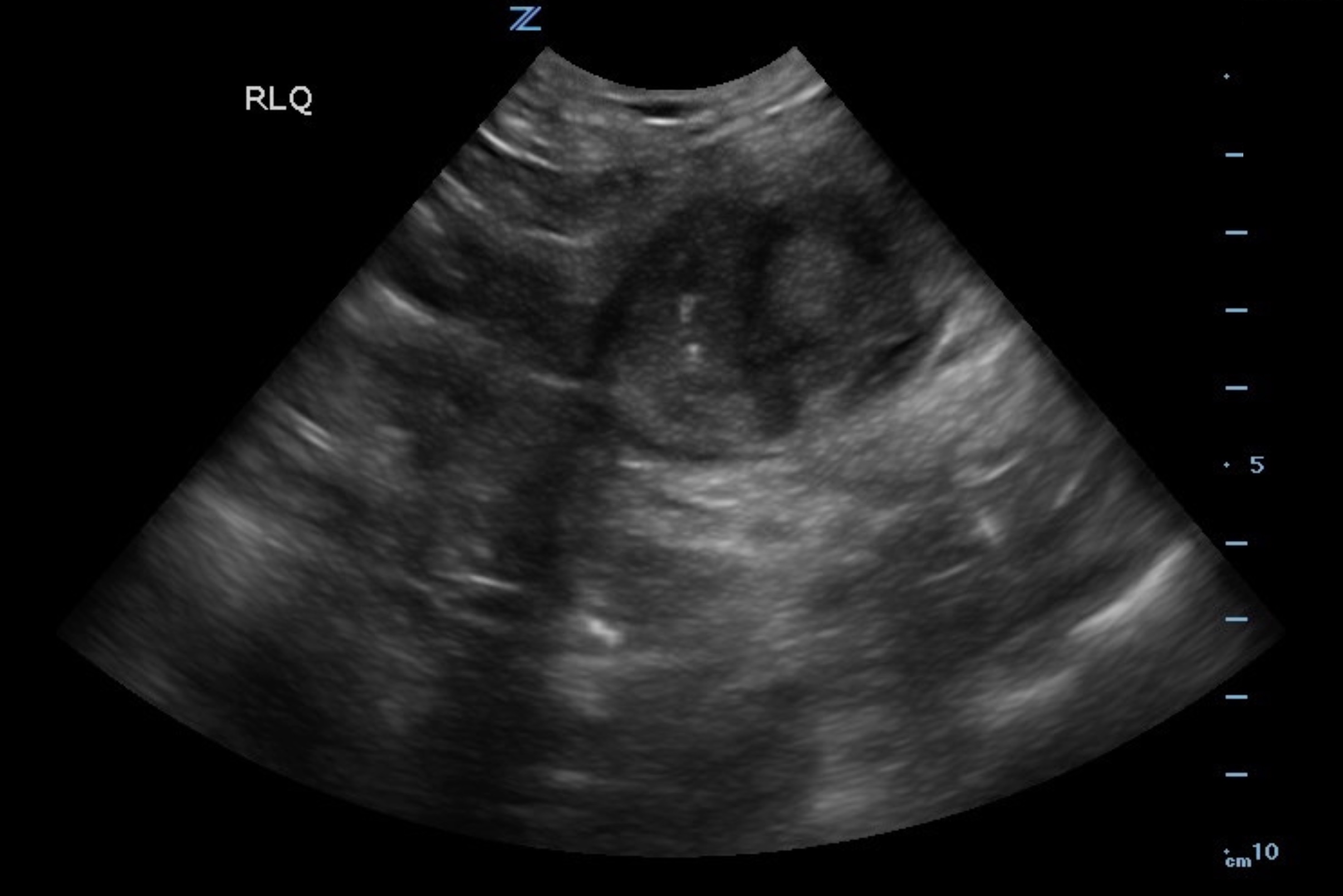

Supplement: Supplementary file 2 [file jetem-8-1-v1-supp2.jpg]

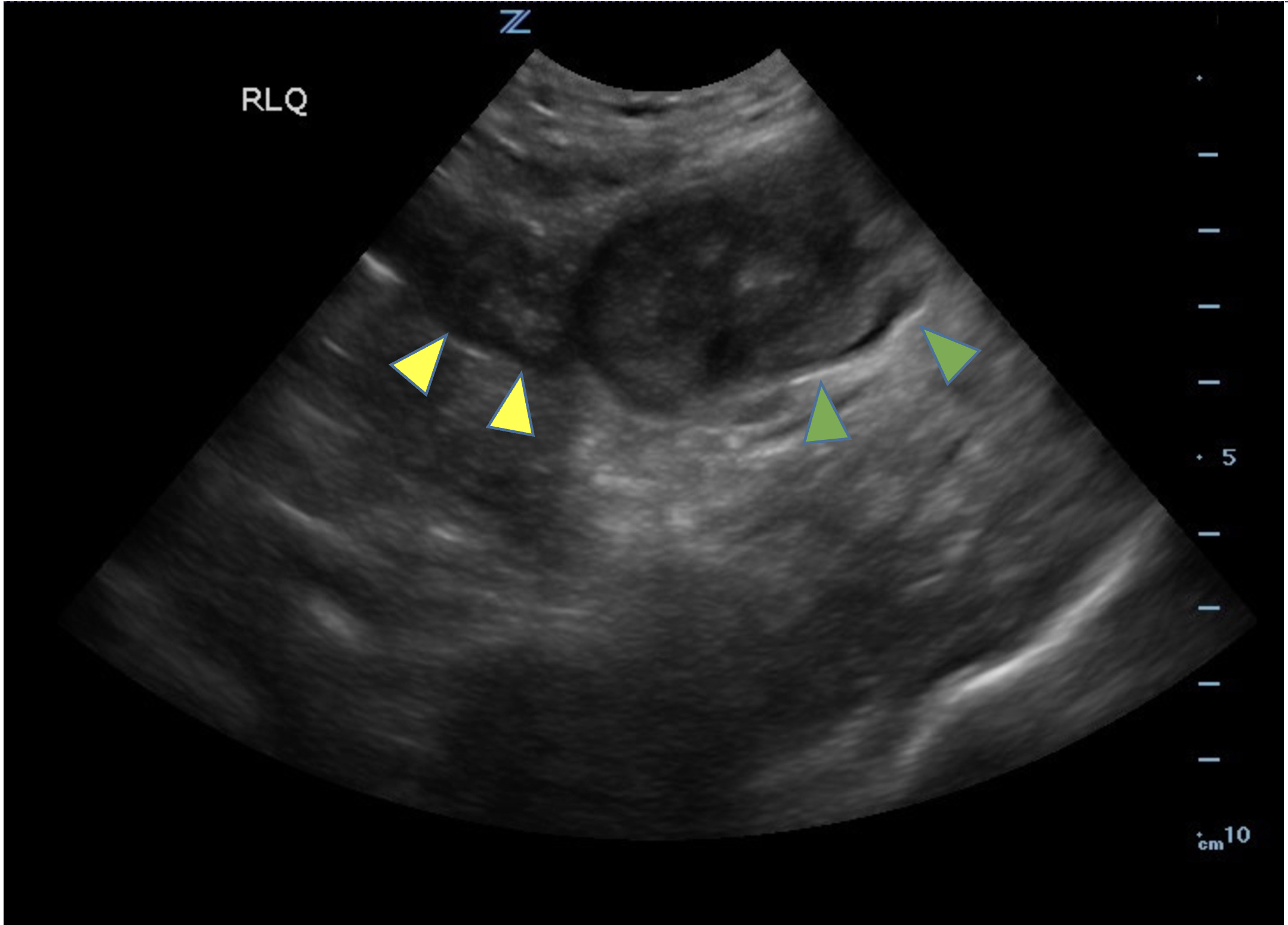

Supplement: Supplementary file 3 [file jetem-8-1-v1-supp3.jpg]

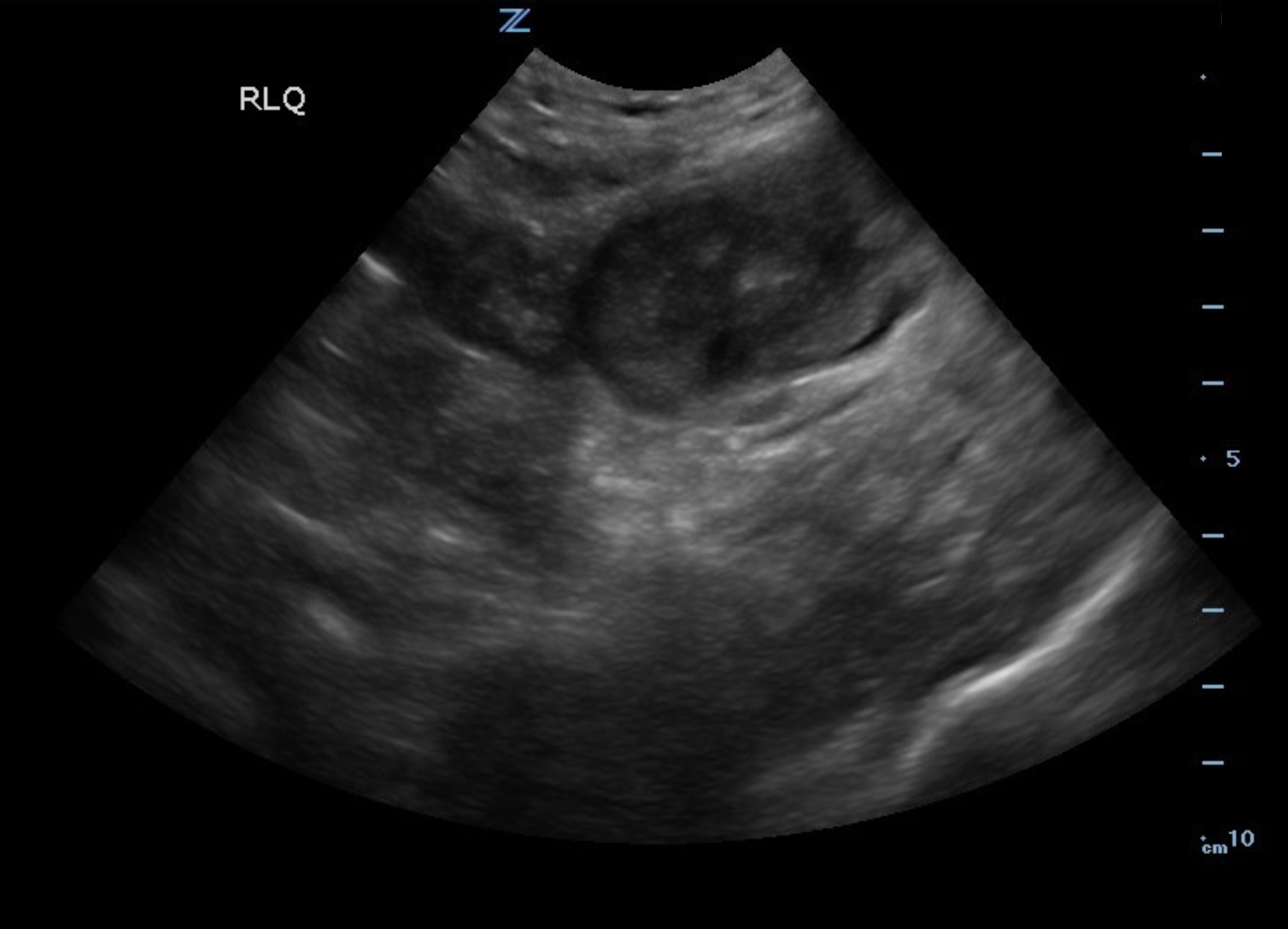

Supplement: Supplementary file 4 [file jetem-8-1-v1-supp4.jpg]

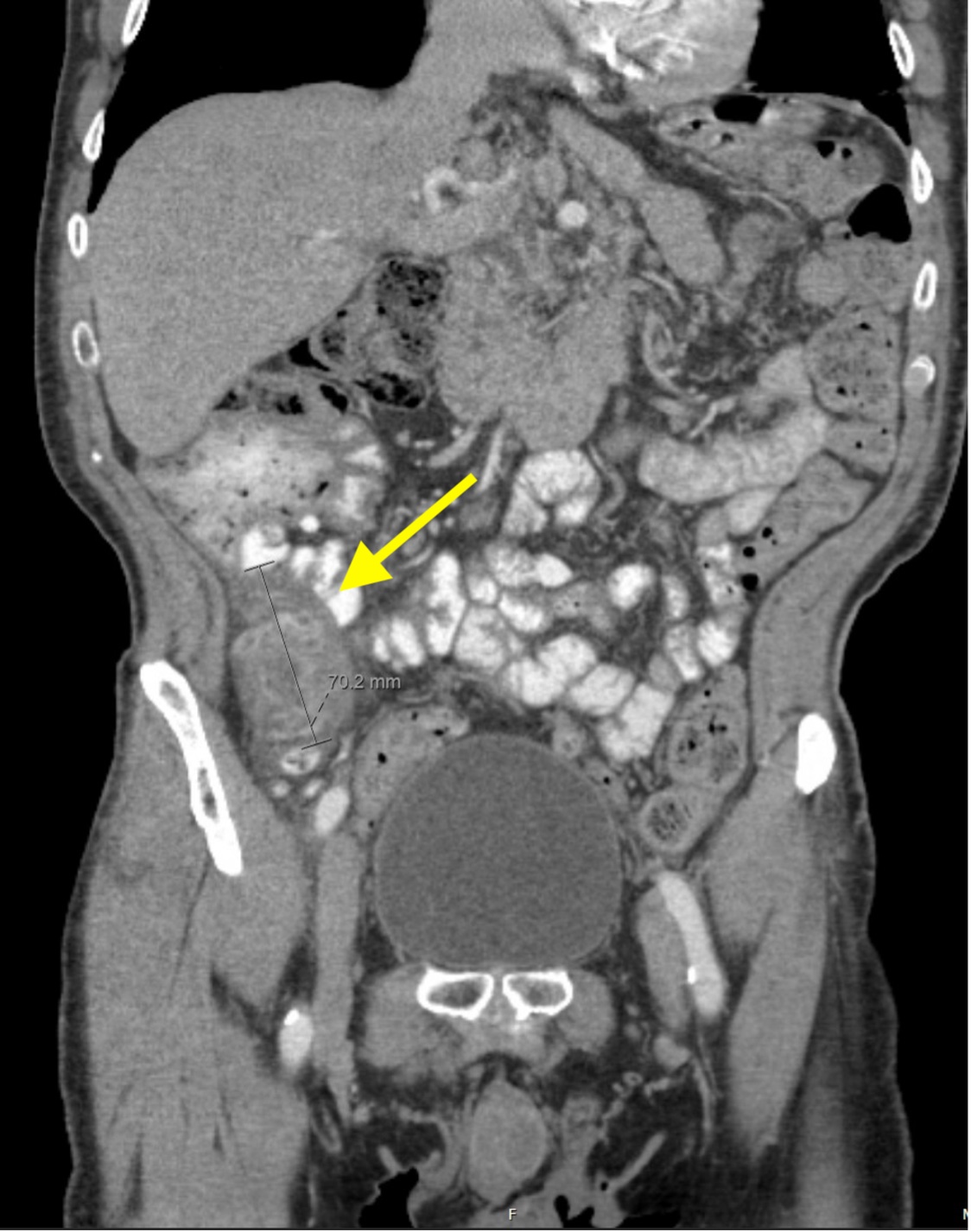

Supplement: Supplementary file 5 [file jetem-8-1-v1-supp5.jpg]

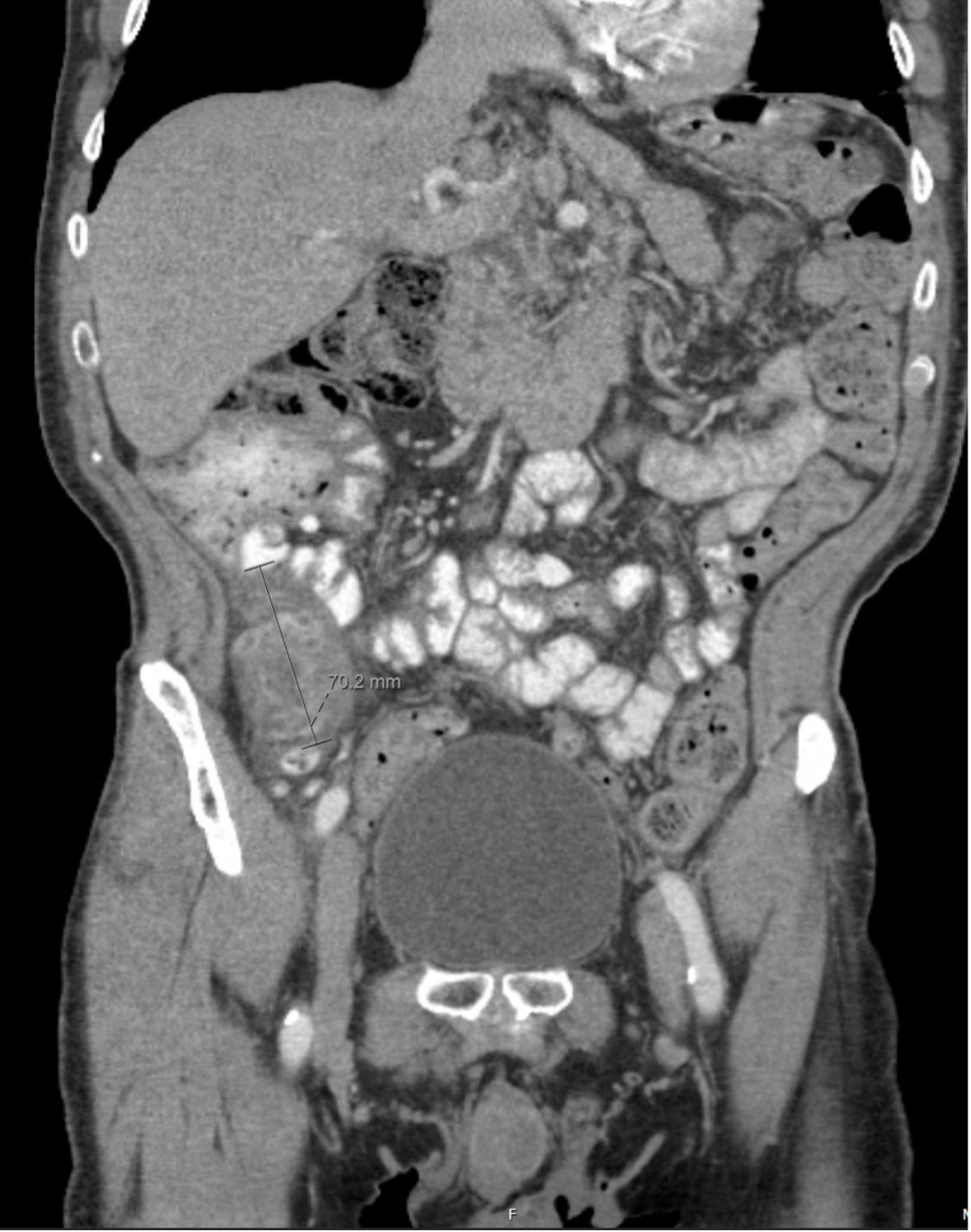

Supplement: Supplementary file 6 [file jetem-8-1-v1-supp6.jpg]

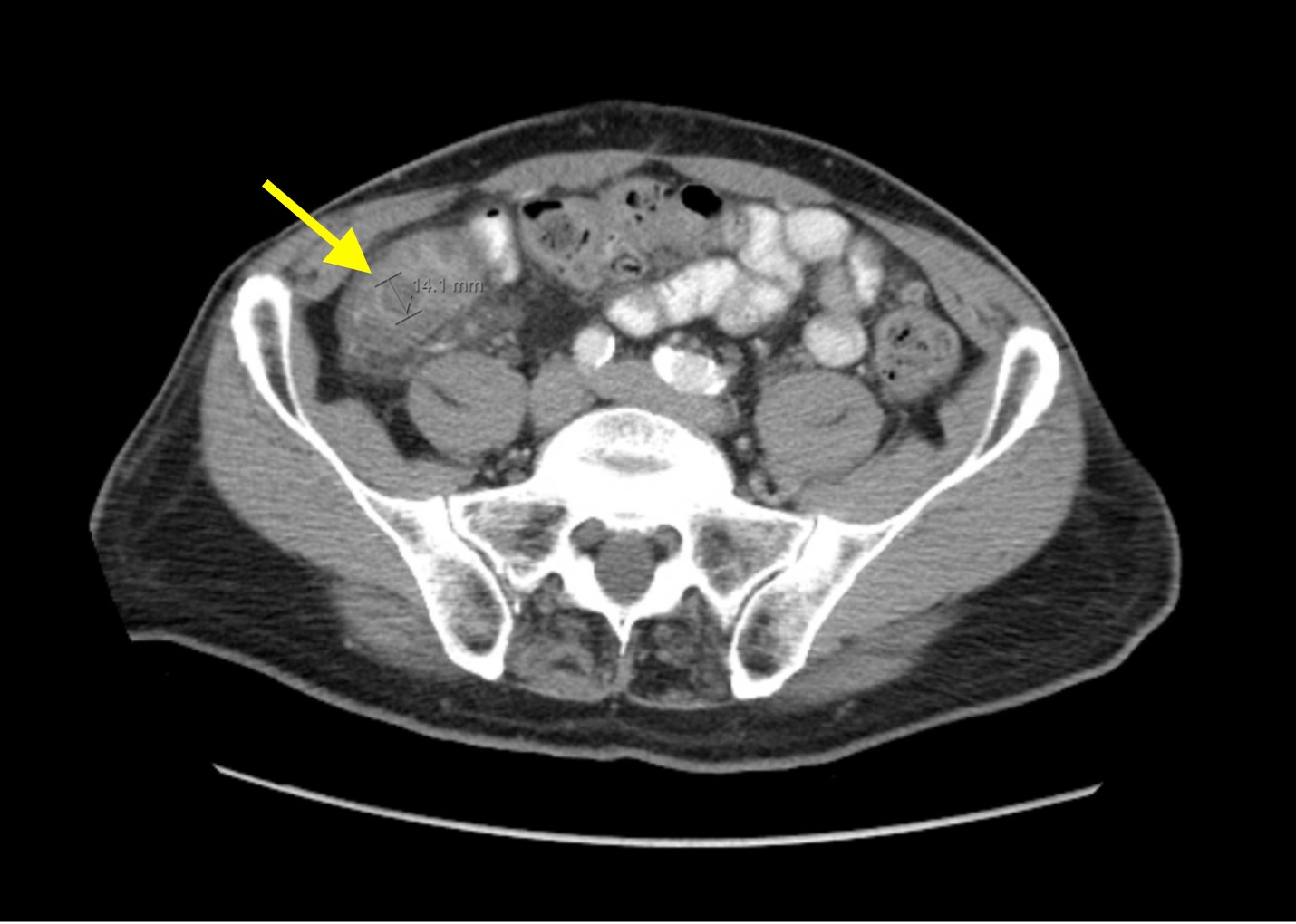

Supplement: Supplementary file 7 [file jetem-8-1-v1-supp7.jpg]

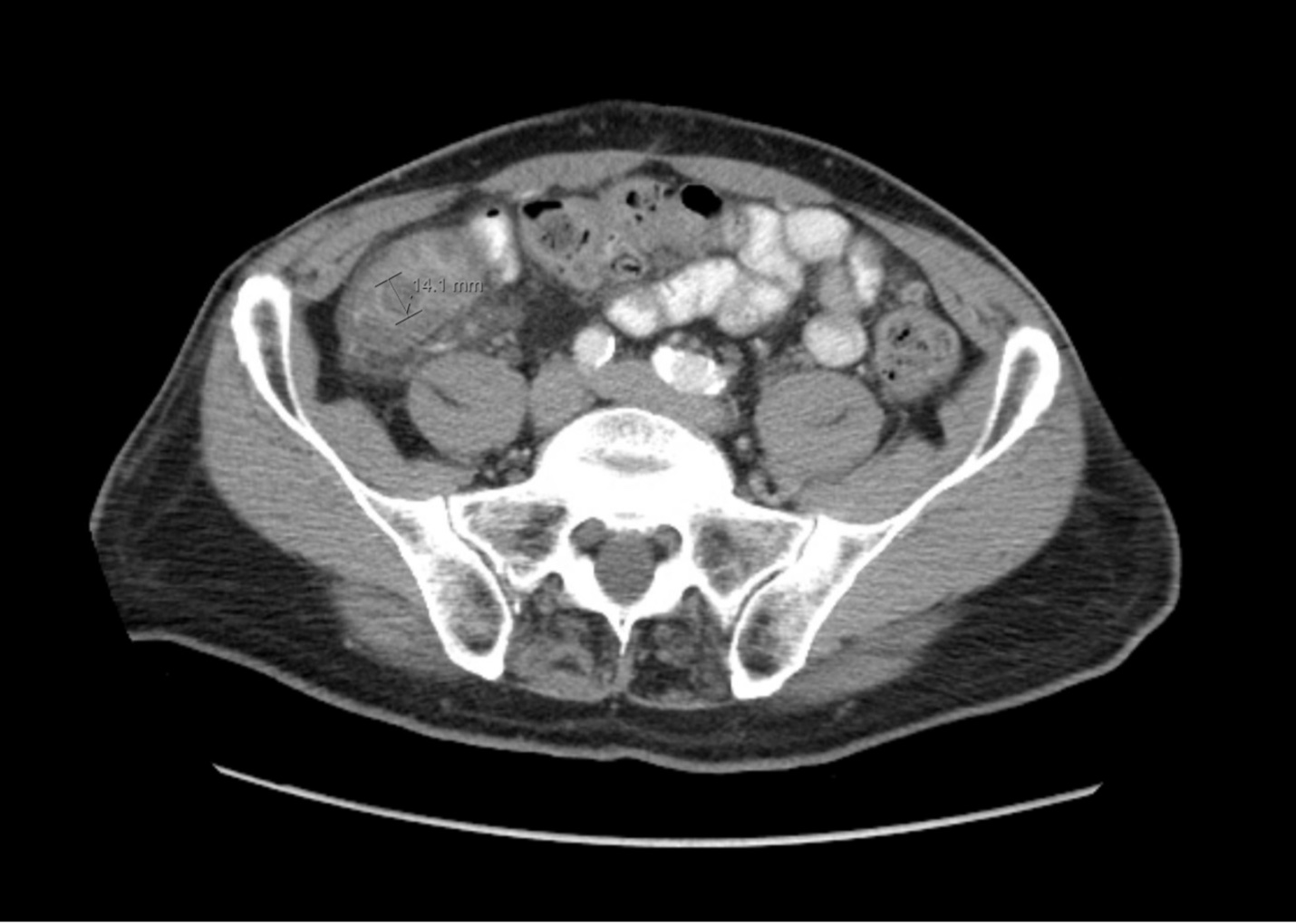

Supplement: Supplementary file 8 [file jetem-8-1-v1-supp8.jpg]
